# The phosphoproteome in regenerating protoplasts from *Physcomitrella patens* protonemata shows changes paralleling postembryonic development in higher plants

**DOI:** 10.1093/jxb/eru082

**Published:** 2014-04-03

**Authors:** Xiaoqin Wang, Meiyan Qi, Jingyun Li, Zhongzhong Ji, Yong Hu, Fang Bao, Ramamurthy Mahalingam, Yikun He

**Affiliations:** ^1^Key Laboratory of Urban Agriculture (North) Ministry of Agriculture, Beijing University of Agriculture, Beijing 102206, China; ^2^College of Life Sciences, Capital Normal University, Beijing 100048, China; ^3^Department of Biochemistry and Molecular Biology, Oklahoma State University, OK 74078, USA

**Keywords:** LC-MS/MS, *Physcomitrella patens*, phosphoproteome, postembryonic development, protoplast regeneration, TiO_2_ enrichment.

## Abstract

The moss *Physcomitrella patens* is an ideal model plant to study plant developmental processes. To better understand the mechanism of protoplast regeneration, a phosphoproteome analysis was performed. Protoplasts were prepared from protonemata. By 4 d of protoplast regeneration, the first cell divisions had ensued. Through a highly selective titanium dioxide (TiO_2_)-based phosphopeptide enrichment method and mass spectrometric technology, more than 300 phosphoproteins were identified as protoplast regeneration responsive. Of these, 108 phosphoproteins were present on day 4 but not in fresh protoplasts or those cultured for 2 d. These proteins are catalogued here. They were involved in cell-wall metabolism, transcription, signal transduction, cell growth/division, and cell structure. These protein functions are related to cell morphogenesis, organogenesis, and development adjustment. This study presents a comprehensive analysis of phosphoproteome involved in protoplast regeneration and indicates that the mechanism of plant protoplast regeneration is similar to that of postembryonic development.

## Summary

During protoplast regeneration, proteins related to cell morphogenesis, organogenesis and development adjustment were phosphorylated in *Physcomitrella patens*. These proteins play important roles in regulating postembryonic development in higher plants.

## Introduction

Plant leaf mesophyll cells can be separated from their original tissue by cell-wall-degrading enzymes generating a large population of protoplasts that can then become totipotent and hence regenerate whole plants (Zhao *et al.*, 2001). Becoming totipotent involves changes in DNA methylation pattern and increased transcription and reorganization of specific chromosomal subdomains (Zhao *et al.*, 2001; Avivi *et al.*, 2004), changes that are similar to those during embryo development, implying a similar mechanism. Protoplasts are also used to observe cellular processes and activities, such as cell-wall synthesis, cell cycle, and differentiation during regeneration, and hormone responses in various plant species (Sheen, 2001). These cellular processes might be similar to plant postembryonic development.

In plants, postembryonic development is organized by meristems, which both self-renew and produce daughter cells that differentiate and give rise to different organ structures. Mechanisms mediating postembryonic development have been mainly studied in seed plants. It has been established that the cell wall is responsible for organ shape and that the cytoskeleton plays an important role in cell division and expansion. Additionally, cell-cycle regulation is essential for development. Some cell-cycle regulators, such as cyclins and cyclin-dependent kinases, are particularly numerous in plants, reflecting the remarkable ability of plants to modulate their development (Inze and De Veylder, 2006).

Organogenesis is a postembryonic process that occurs in a continuous manner throughout the entire lifespan. During organogenesis, organ identity genes play a key role. In the course of shoot propagation, a number of regulators have been identified, including KNOTTED1, SHOOT MERISTEMLESS (STM), KNOTTED-like from *Arabidopsis thaliana* 2 (KNAT2), and CUP-SHAPED COTYLEDON 1 and 2 (CUC1 and CUC2) (Vollbrecht *et al.*, 1991; Long *et al.*, 1996; Pautot *et al.*, 2001; Cary *et al.*, 2002; Gordon *et al.*, 2007). In contrast, for root formation, certain other proteins appear to play dominant roles, including the PINFORMED (PIN) family, transport inhibitor response1 (TIR1), and the Aux/IAA family of transcription factors (Geldner *et al.*, 2001; Rogg *et al.*, 2001; Friml *et al*., 2002a, b). During organogenesis, DNA methylation, histone methylation and acetylation are important for the enormous variation in cell-type-specific and stage-specific gene expression (Fransz and de Jong, 2002). However, much less is known the molecular mechanism of protoplast regeneration.

The moss *Physcomitrella patens* has been established as a model system for the study of plant development (Cove *et al.*, 1997; Sakakibara *et al.*, 2003). As with all plants, the form of the moss plant is determined by the pattern of growth and division. Protoplast cultures provide an ideal system for the study of development because following protoplast formation intact plants are produced at high frequency and rapidly. In protoplasts formed from seed plants, the process of regeneration has been associated with numerous events, including dedifferentiation and the loss of photoautotrophic metabolism (Fleck *et al.*, 1980; Vernet *et al.*, 1982; Criqui *et al.*, 1992; Nagata *et al.*, 1994), cell-wall synthesis (Meyer and Abel, 1975), and activation of the cell-cycle machinery (Galbraith *et al.*, 1981, 1983). The cell cycle is regulated by key developmental regulators, which are themselves phosphoregulated (Joubes *et al.*, 2000).

Protein phosphorylation is among the most important post-translational modifications in cells. It underlies many regulatory functions, such as cell-cycle control, receptor-mediated signal transduction, differentiation, and metabolism. For these regulatory functions, eukaryotic cells rely extensively on phosphorylating the hydroxyl group of the side chains of serine, threonine, and tyrosine (Hunter, 1995; Schlessinger, 2000).

Here, phosphoproteomics has been used to increase understanding of the machinery of protoplast regeneration in *P. patens*. The work examined the global changes in the phosphoproteome following protoplast development using titanium dioxide (TiO_2_) phosphopeptide enrichment strategies coupled with LC-MS/MS. The study reveals the integration of protoplast regeneration mechanisms in *P. patens*.

## Materials and methods

### Plant material and harvesting

*P. patens* (Hedwig) ecotype ‘Gransden 2004’ was grown in BCDA medium as described (Khandelwal *et al.*, 2010). Protonemata were cultured at 25 °C under a 16/8 light/dark cycle. To produce protoplasts, 7-d-old protonemata were treated for 1h with 0.5% driselase dissolved in 8% mannitol. After filtration and washing, the protoplasts are regenerated on liquid BCDA medium with 8% mannitol under the same culture conditions. Protoplast isolation was repeated three times. For analysis of the next experiment, freshly prepared protoplasts and those cultured for 2 and 4 d were harvested.

### Fluorescence-activated cell sorter analysis

For fluorescence-activated cell sorter (FACS) analysis, nuclei were stained with 2.86 μM 2-(4-amidinophenyl)-6-indolecarbamidine dihydrochloride (DAPI) and analysed on a two-laser FACStar Plus platform (Becton Dickinson, Mountain View, CA, USA). An argon ion laser tuned to 488nm was used in the laser experiment and a detector with a 530 band pass filter was used for fluorescein isothiocyanate. Software compensation was applied to the collected data using CELLQUEST software (Becton Dickinson).

### Protein extraction

Frozen plant material was suspended in 2ml extraction buffer containing 250mM sucrose, 20mM Tris-HCl (pH 7.5), 10mM EDTA, 1mM 1,4-dithiothreitol (DTT), and inhibitor cocktail for proteases (Sigma) and phosphatases (Sigma). Cell debris was removed by centrifugation at 8000 *g* for 10min at 4 °C. Supernatants were transferred to clean tubes and centrifuged at 120 000 *g* for 1h at 4 °C. The final supernatants were used for soluble protein extraction, as described previously (Wang *et al.*, 2009). The pellets (membrane-associated proteins) were re-precipitated with acetone overnight at –20 °C. The precipitated membrane proteins were rinsed three times with ice-cold acetone containing 13mM DTT and subsequently lyophilized. Finally, both protein pellets were resuspended separately by adding 8M urea, 4% CHAPS, 65mM DTT, and 40mM Tris (pH 7.5). The protein concentration was determined according to Peterson (1977) using BSA as a standard. The supernatants were stored in aliquots at –80 °C or directly digested with trypsin.

### Tryptic digestion and phosphopeptide purification

The tryptic digestion was processed as described previously (Dai *et al.*, 2005). In brief, 500 μg of each protein sample (100 μl volume) were reduced with 20mM DTT at 37 °C for 2.5h and alkylated with 100mM iodoacetamide for 40min at room temperature in the dark. After that, the protein mixtures were spun and exchanged into 100mM ammonium bicarbonate buffer (pH 8.5), and then incubated at 37 °C for 16h with trypsin at an enzyme/substrate ratio of 1:50 (w/w) to produce a proteolytic digest. Finally, the digested peptide mixture was lyophilized and then diluted in a loading buffer containing 1M glycolic acid in 65% acetonitrile (ACN) and 2% trifluoroacetic acid (TFA).

Phosphopeptide purification was performed using TiO_2_ microcolumns (320 μm×50mm, Column Technology, Freemont, CA, USA; Thingholm and Larsen, 2009). The microcolumns were rinsed with 100 μl loading buffer and the samples (200 μl) were subsequently loaded by applying air pressure. After loading the sample onto the microcolumn, the columns were subsequently washed with 100 μl loading buffer, 100 μl washing buffer I (65% ACN and 0.5% TFA), and 100 μl washing buffer II (65% ACN and 0.1% TFA). The bound peptides were eluted with 100 μl elution buffer (300mM ammonium water and 50% ACN). The elution was acidified by adding 5 μl 100% formic acid prior to the desalting step.

### Nano-LC/MS/MS analysis

Peptide separation was performed on a surveyor liquid chromatography system (Thermo Finnigan, San Jose, CA, USA), consisting of a degasser, MS pump, and autosampler and equipped with a C18 trap column (RP, 320 μm×20mm, Column Technology) and an analytical C18 column (RP, 75 μm×150mm, Column Technology). After sample loading, the column was washed for 30min with 98% mobile phase A (0.1% formic acid in water) to flush off remaining salt. Peptides were eluted using a linear gradient of increasing mobile phase B (0.1% formic acid in ACN) from 2 to 35% in 120min. A linear ion trap/Orbitrap hybrid mass spectrometer (Thermo Fisher, San Jose, CA, USA) equipped with a NSI nanospray source was used for the MS/MS experiment. Spray voltage applying to the Nano needle was 1.85kV and ion transfer capillary temperature was 160 °C. Normalized collision energy for collision-induced dissociation was 35%. The number of ions stored in the ion trap was regulated by the automatic gain control. The instrument method consisted of one full MS scan from 400 to 2000 m/z followed by data-dependent MS/MS scan of the 10 most-intense ions from the MS spectrum with the following dynamic exclusion settings: repeat count of 2, repeat duration 30 s, exclusion duration 1.5min. The resolution of the Orbitrap mass analyser was set at 100 000 (m/Δm 50% at m/z 400) for the precursor ion scans.

### Protein assignment

The strategy for identifying phosphorylated proteins in *P. patens* was as follows. The MS/MS spectra files from each LC run were centroided and merged to a single file using the TurboSEQUEST program in the BioWorks 3.2 software suite (Thermo Electron), and then the MS/MS spectra were searched against the NCBI *A. thaliana* and *P. patens* combined protein database (including normal and reversed) with carbamidomethylcysteine as a fixed modification. Oxidized methionine and phosphorylation (serine, threonine, and tyrosine) were searched as variable modifications. The searches were performed with tryptic specificity allowing one missed cleavage and the precursor ion m/z tolerances of 50 ppm and fragment ion m/z tolerances of ±1Da. Cysteine residues were searched as a fixed modification by 57.02146Da because of carboxyamidomethylation. Oxidation was set as a variable modification on methionine (15.99492Da). Dynamic modifications were permitted to allow for the detection of phosphorylated serine, threonine, and tyrosine residues (+79.96633). The phosphoric acid neutral loss peaks of serine and threonine was about –18.01056Da.

To provide high-confidence phosphopeptide sequence assignments, an accepted SEQUEST result had to have a Δ*C*_n_ score of at least 0.1 (regardless of charge state). Peptides with a +1 charge state were accepted if they were fully digested and had a cross correlation (Xcorr) of at least 1.9. Peptides with a +2 charge state were accepted if they had a Xcorr ≥2.2. Peptides with a +3 charge state were accepted if they had a Xcorr ≥3.3. All output results were filtered and combined together using BuildSummary software to delete the redundant data (Tabb *et al.*, 2002). All identified proteins (whether phosphorylated or not) were calculated separately and filtered by precursor ion tolerance m/z of 10 ppm and 1.0% false-positive rate (Elias and Gygi, 2007). The false-positive rate (FPR) was calculated as: FPR = 2[*N*_rev_/(*N*_rev_ + *N*_for_)], where *N*_rev_ is the number of hits to the ‘reverse’ peptide and *N*_for_ is the number of hits to the ‘forward’ peptide. The proteins were classified to a protein group if the same peptides were assigned to multiple proteins after false peptides were filtered. A further manual check removed phosphopeptides with unclear MS/MS spectra.

For phosphorylation site identification, first a stricter peptide identification criteria (FPR≤0.01) was set. Second, a modified site was considered to be unique only when the corresponding modified peptides had a Δ*C*_n_ >0.1 because a Δ*C*_n_ >0.1 is significant for discriminating the first (top) candidate peptide from the second candidate peptide (Deng *et al.*, 2010). In addition, this work checked the phosphoric acid neutral loss peaks for phosphorylation site identification (Ballif *et al.*, 2004).

### Analysis of gene expression by real-time reverse-transcription PCR

Total RNA was extracted using a RNeasy Plant Mini Kit (Qiagen, Hilden, Germany). After extraction, RNA samples were treated with DNase (Ambion, USA). First-strand cDNA was synthesized from total RNA using a iScript cDNA Synthesis Kit (Bio-Rad, Hercules, CA, USA) according to the manufacturer’s instructions. The *P.* *patens* actin3 cDNA gene was used as a standard to normalize the content of cDNA. Real-time reverse-transcription PCR was performed using gene-specific primers for phosphoproteins in the *P*. *patens* protein database and phosphoproteins in the *A*. *thaliana* protein database that had genes homologous to those in the *P*. *patens* database (Supplementary Tables S1 and S2, respectively, available at *JXB* online) on a Rotor Gene 3000 Real-Time Thermal Cycler (Corbet Research, Australia). SYBR Premix Ex Taq (Perfect Real Time) kit and reverse-transcription PCR reagents (Takara Bio) were used for quantification of differentially expressed gene sequences.

## Results

### Protoplast cell-cycle phase

**Fig. 1.**
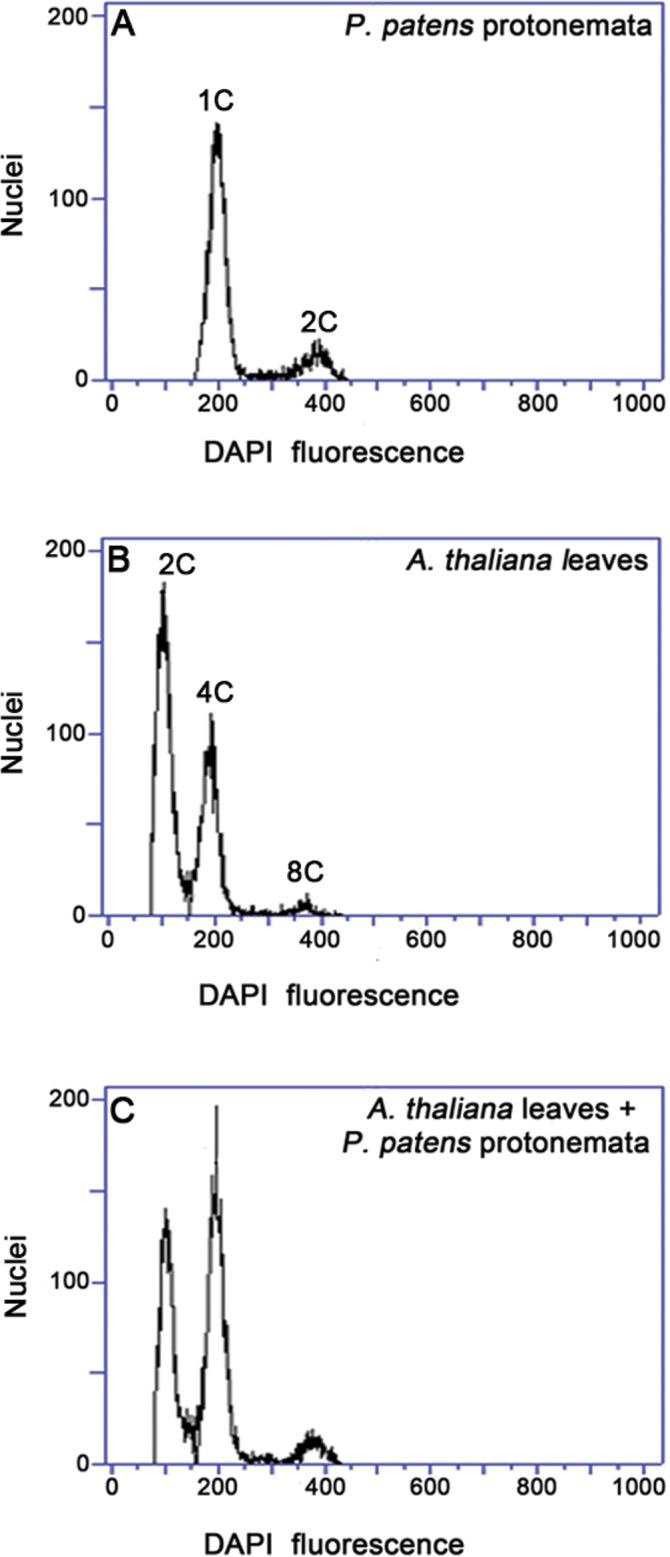
Identification of cell-cycle phases. Nuclei were prepared from *Physcomitrella patens* protonemata (A) and *Arabiposis thaliana* leaves (B) or a mixture of nuclei from both species (C), stained with DAPI, and subjected to FACS analysis.

To identify the phase of the cell cycle for cells in *P. patens* protonemata, the DNA content of protonemata cell nuclei was measured with FACS. The standard phase of cell cycle was determined using nuclei from *A. thaliana* leaves. The nuclei from *A. thaliana* had three peaks and two peaks from *P. patens*, and the second peak in *A. thaliana* has approximately the same relative fluorescence value as that of *P. patens* protonemata in the first peak (Fig. 1A and B). The *A. thaliana* genome size is 125Mb and the leaves are diploid. The *P. patens* protonemata are haploid and its genome size is 490Mb. The nuclei in the second peak of *A. thaliana* leaves are in G2 phase (4C, 500Mb; Fig. 1B). So, it was speculated that the nuclei from *P. patens* protonemata were in G1 phase (1C, 490Mb; Fig. 1A).

**Fig. 2.**
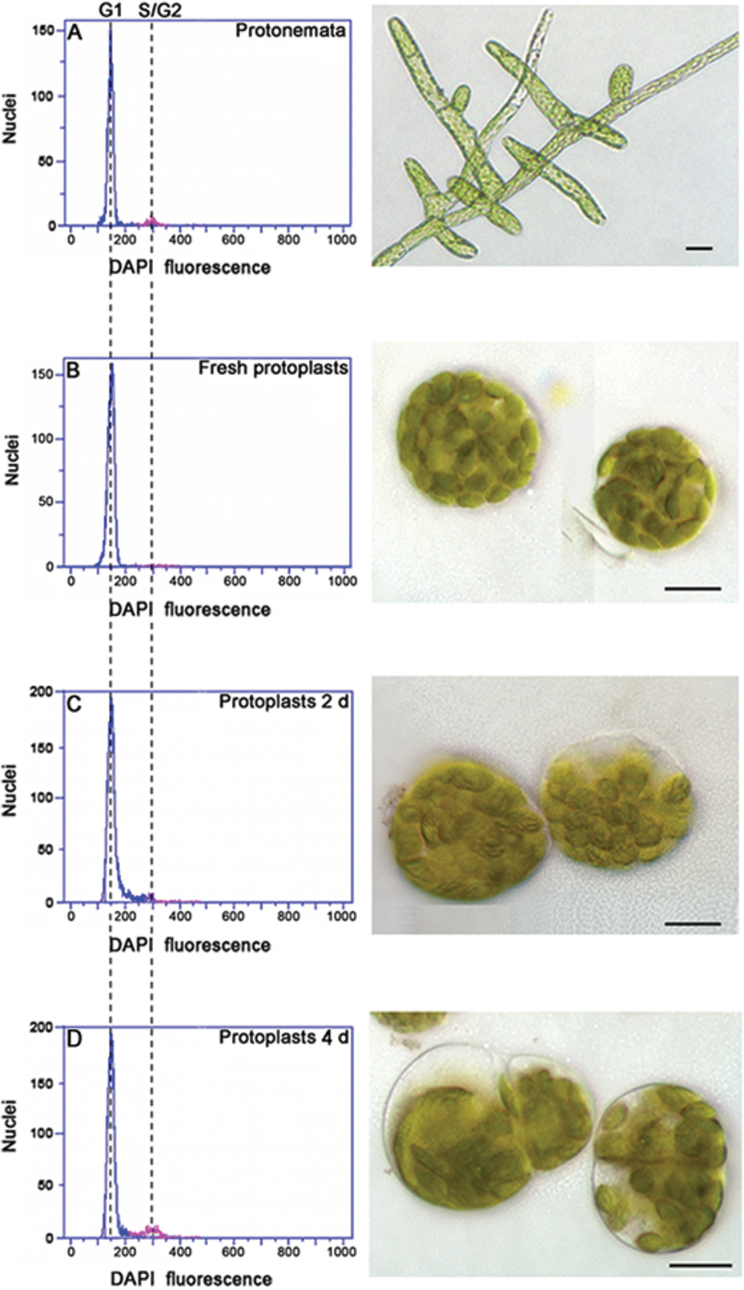
Protoplast regeneration and the cell cycle. (A) Protonemata. (B) Protoplasts. (C) Protoplasts after regeneration for 2 d. (D) Protoplasts after regeneration for 4 d. Nuclei were isolated from *Physcomitrella patens* protonemata or protoplasts at the indicated times, stained with DAPI, and subjected to FACS analysis. Bright-field images show representative cells. Bars, 20 μm.

To investigate how *P. patens* protoplasts regenerate, 7-d-old protonemata were used to establish an efficient and reproducible ‘protoplast system’. FACS analysis showed that most protonemal nuclei (92%) had a DNA content corresponding to G1 phase and a small peak (8%) was present at a S/G2 level (Fig. 2A), whereas nearly 100% of the nuclei from freshly harvested protoplasts had a G1 level of DNA (Fig. 2B). This is consistent with previous report. Tobacco leaves were treated with cell-wall-degrading enzymes to produce a large population of protoplasts, which had a DNA content corresponding to G1 phase (Zhao *et al.*, 2001). Fresh protoplasts appeared round and green. By 2 d of regeneration, the new polar axes were established and protoplasts with a S/G2 level of DNA were present (constituting about 8% of the population; Fig. 2C). By 4 d, asymmetric cell divisions were common and protoplasts with a S/G2 DNA content constituted about 13% of the population (Fig. 2D). Subsequently, the cultures were transferred to a regeneration medium (BCDA medium) for formation of protonemata.

### Phosphopeptide enrichment and LC-MS/MS identification

To analyse the *P. patens* phosphoproteome, this work used a TiO_2_ phosphopeptide enrichment strategy in combination with LC-MS/MS for identification. The resulting data were analysed using the TurboSEQUEST program in the BioWorks 3.2 software suite. From the three treatments altogether, more than 2000 phosphoproteins were identified (data not shown). This work focused on phosphoproteins in protoplasts regenerated for 4 d. There were more than 300 of these expressed in protoplasts regenerated for 4 d which were not present in fresh protoplasts or those regenerated for 2 d. Of this group of unique phosphoproteins, 108 of them were functional annotation proteins and others are predicted proteins. These 108 phosphoproteins were chosen for further analysis (Table 1).

**Table 1.** Phosphoproteins in *Physcomitrella patens* unique to protoplast regenerated 4 d* indicates methionine oxidation; # indicates phosphorylation sites.

Metabolic group	Protein no.	Theoretical mass (kDa)	Theoretical pI	Accession no.	Description	Subcellular localization	Sequence
Metabolism (19.44%)	C1	32.1	5.05	NP_194311	XTR6 probable xyloglucan endotransglucosylase/ hydrolase protein 23	Cell wall	M*Y#S#SLWNAEEWATR
	C2	61.9	9.08	NP_187947	Sks11 (SKU5 Similar 11); copper ion binding/oxidoreductase	Membrane	NCWQDGT#PGT#MCPIMPGT#NYTYHFQPK
	C3	116.4	5.96	NP_172637	ATSS3 (starch synthase 3); starch synthase/transferase, transferring glycosyl groups	Chloroplast	DS#NATST#ATNEVSGISK
	C4	42.2	5.5	NP_001031705	CORI3 (CORONATINE INDUCED 1); cystathionine beta-lyase/transaminase	Apoplast	FSSIVPVVT#LGSISK
	C5	152.4	5.77	NP_193817	TPP2 (TRIPEPTIDYL PEPTIDASE II); tripeptidyl-peptidase	Unknown	VLDVIDCT#GSGDIDTST#VVK
	C6	72.1	6.31	Q8RXN5	tRNA wybutosine-synthesizing protein 1 homologue	Unknown	CMET#T#PS#LACANK
	C7	44.0	7.54	NP_568193	AtAGAL2 (*Arabidopsis thaliana* ALPHA-GALACTOSIDASE 2); alpha-galactosidase/catalytic/hydrolase, hydrolysing *O*-glycosyl compounds	Cell wall	FIAFT#LTITLT#QIADGFQS#R
	C8	61.5	8.93	NP_196950	Pseudouridine synthase/transporter	Chloroplast	GET#VELS#PR
	C9	7.04	6.05	Q9C6T3	Terpene synthase family protein, putative	Membrane	LIHLLVS#M*GISYHFDK
	C10	66.6	6.63	NP_191966	Malate oxidoreductase, putative	Mitochondrion	MAGIS#ES#EATK
	C11	87.6	6.37	Q9FZI2	Lupeol synthase 5	Unknown	CCMLLS#T#M*PTDITGEK
	C12	38.3	4.61	NP_849593	Lipid-binding serum glycoprotein family protein	Membrane	DQIGS#SVEST#IAK
	C13	40.1	8.87	NP_196027	Short-chain dehydrogenase/reductase (SDR) family protein	Unknown	FNHS#HT#PVCVITGAT#SGLGK
	C14	60.2	8.74	Q8W033	Aldehyde dehydrogenase family 3 member I1	Chloroplast	LFS#EYLDNTT#IR
	C15	79.9	6.12	NP_566041	Transketolase, putative	Chloroplast	S#IGIDT#FGASAPAGK
	C16	45.8	5.12	NP_196694	Monooxygenase family protein	Membrane	S#SEPPVGSPT#GAGLGLDPQAR
	C17	52.3	5.6	Q9C7Z9	Serine carboxypeptidase-like 18	Membrane	T#EGYNGGLPSLVSTSYS#WTK
	C18	39.7	5.27	NP_197540	Oxidoreductase, 2OG-Fe(II) oxygenase family protein	Unknown	SLELEENSFLDM*Y#GESATLDT#R
	C19	25.8	8.79	Q8VY52	PsbP domain-containing protein 2	Chloroplast	FFCFAQNPS#S#TVSINLSK
	C20	134.7	7.39	NP_196999	GMII (GOLGI ALPHA-MANNOSIDASE II); alpha-mannosidase	Golgi apparatus	PLNDS#NS#GAVVDITT#K
	C21	57.6	9.09	NP_193230	ATAO1 (ARABIDOPSIS THALIANA AMINE OXIDASE 1); amine oxidase/copper ion binding	Cell wall	VGVDLT#GVLEVK
Transcription (22.22%)	C22	62.5	7.72	NP_192756	Light-mediated development protein DET1	Nucleus	FGLFATSTAQIHDSS#SPS#NDAVPGVPS#IDK
	C23	137.4	9.28	NP_568624	COP1-interacting protein-related	Membrane	VYT#LETEIIQIK
	C24	102.4	8.35	EDQ54570.1	Trithorax-like protein, histone–lysine *N*-methyltransferase	Nucleus	TDCLTY#QPPVT#S#SGCAR
	C25	70.3	4.44	EDQ52478.1	Paf1 complex protein	Nucleus	GLSPGY#LEDALEEEDEPDQYDR
	C26	110.5	4.91	EDQ60631.1	Putative histone deacetylase complex, SIN3 component	Nucleus	FM*QVLY#GLLDGSVDNSK
	C27	72.4	8.63	NP_181971	DNA-binding bromodomain-containing protein	Nucleus	SMNESNST#ATAGEEER
	C28	35.6	4.91	P46640	KNAT2 homeobox protein knotted-1-like 2	Nucleus	AEDNFS#LSVIK
	C29	59.9	6.96	NP_199123	APUM13 (*Arabidopsis* Pumilio 13); RNA binding/binding	Nucleus	IMDAISSVALQLT#R
	C30	29.5	8.75	EDQ59312.1	Type I MADS-domain protein PPTIM4	Nucleus	MS#TT#TSPPALT#VK
	C31	114.8	8.71	NP_173522	Squamosa promoter-binding-like protein 14	Nucleus	GLDLNLGS#GLT#AVEETTTTTQNVR
	C32	40.2	5.70	NP_198791	AGL81 (AGAMOUS-LIKE 81); transcription factor	Nucleus	CSSS#SSS#SSYSLASTSLSNR
	C33	19.9	6.21	NP_001078798	AGL31 (AGAMOUS LIKE MADS-BOX PROTEIN 31); transcription factor	Unknown	TS#LEANSS#VDTQ
	C34	64.9	5.02	NP_179283	AMS (ABORTED MICROSPORES); DNA binding/transcription factor	Nucleus	LMEALDS#LGLEVT#NANTTR
	C35	176.3	5.65	EDQ61350.1	SNF2 family DNA-dependent ATPase	Nucleus	S#PENGS#PAQEYDSPS#R
	C36	108.1	6.84	NP_564568	SNF2 domain-containing protein/helicase domain-containing protein/RING finger domain-containing protein	Nucleus	LDGT#MS#LIAR
	C37	200.2	8.50	EDQ61497.1	SNF2 family chromodomain-helicase	Nucleus	NY#QEEGVTWLDHNFENR
	C38	102.1	9.52	EDQ66080.1	Argonaute family member	Unknown	LMSPLEGGSTTS#SS#SS#R
	C39	42.5	5.40	NP_179397	VND1 (VASCULAR RELATED NAC-DOMAIN PROTEIN 1); transcription factor	Unknown	FVASQLMS#QEDNGTS#SFAGHHIVNEDK
	C40	34.6	5.47	NP_190248	PCL1 (PHYTOCLOCK 1); DNA binding/transcription factor	Unknown	FGSMAS#Y#PSVGGGS#ANEN
	C41	263.8	5.34	NP_171710	Transcriptional regulator-related	Membrane	ETGT#SLQTLT#SAATM*ER
	C42	67.5	8.98	NP_175971	Transcription factor-related	Unknown	HFWSSY#PITTT#Y#LHTK
	C43	42.8	5.39	NP_566737	S1 RNA-binding domain-containing protein	Chloroplast	DT#S#DEASAAGPSDWK
	C44	36.5	6.91	NP_188063	CID9 (CTC-interacting domain 9); RNA binding/protein binding	Unknown	NFFES#ACGEVTR
	C45	38.2	8.81	NP_567159	DNA-binding storekeeper protein-related	Nucleus	T#LNS#PSAAVAVSDDSES#EK
	C46	36.8	8.33	NP_174084	CYCT1;3 (CYCLIN T 1;3); cyclin-dependent protein kinase	Nucleus	NVSLFESPQCET#S#K
Signal transduction (12.96%)	C47	71.7	6.43	Q8H0V1	CDK5RAP1-like protein	Unknown	AT#HAS#SS#SSSALLPR
	C48	49.6	6.37	NP_568248	CYC3B (MITOTIC-LIKE CYCLIN 3B FROM ARABIDOPSIS); cyclin-dependent protein kinase regulator	Nucleus	WT#LDQTDHPWNPT#LQHYTR
	C49	39.2	9.11	Q9C9M7	CAK4 cyclin-dependent kinase d-2	Cytoplasm	T#IFPM*AS#DDALDLLAK
	C50	134.2	6.53	NP_199264	TAO1 (TARGET OF AVRB OPERATION1); ATP binding/protein binding/transmembrane receptor	Membrane	QFLVDT#EDICEVLTDDT#GT#R
	C51	20.3	6.59	EDQ82848.1	Arf6/ArfB family small GTPase	Unknown	NVNFT#VWDVGGQDK
	C52	33.5	5.72	NP_200984	ATIPK2BETA; inositol or phosphatidylinositol kinase/inositol trisphosphate 6-kinase	Nucleus	LPHLVLDDVVSGY#ANPS#VM*DVK
	C53	21.9	6.84	EDQ74392.1	Sar1 family small GTPase	Unknown	M*GY#GEGFK
	C54	188.8	5.47	NP_195533	Guanine nucleotide exchange family protein	Chloroplast	T#ALGPPPGSS#T#ILSPVQDITFR
	C55	52.5	6.11	NP_001031307	NHO1 (nonhost resistance to P. s. phaseolicola 1); carbohydrate kinase/glycerol kinase	Unknown	AVLES#M*CFQVK
	C56	109.4	5.43	NP_849957	AtRLP19 (receptor-like protein 19); kinase/protein binding	Membrane	LTGTLPSNMSSLS#NLK
	C57	75.5	9.02	NP_565976	TTL3 (TETRATRICOPETIDE-REPEAT THIOREDOXI*N*-LIKE 3); binding/protein binding	Unknown	GSASSSAAATPTS#SSGS#SGSAS#GK
	C58	21.9	6.10	NP_172390	ATSARA1A (ARABIDOPSIS THALIANA SECRETIO*N*-ASSOCIATED RAS SUPER FAMILY 1); GTP binding	Chloroplast	M*GY#GEGFK
	C59	22.6	9.41	NP_196631	Aminoacyl-tRNA hydrolase/protein tyrosine phosphatase	Unknown	FTTAS#PS#M*SQQTGEIDAVVDASSAEK
Transport (10.19%)	C60	96.9	6.92	Q38998	Potassium channel AKT1	Membrane	S#WFLLDLVSTIPSEAAM*R
	C61	19.7	8.86	NP_175324	Plastocyanin-like domain-containing protein	Membrane	VMEVESTPQSPPPS#S#SLPAS#AHK
	C62	50.2	5.65	NP_198698	Amino acid transporter-like protein	Membrane	IMLQVS#ILVSNIGVLIVYM*IIIGDVLAGK
	C63	106.9	5.62	NP_201066	Transportin-SR-related	Nucleus	LDTVTYS#LLALTR
	C64	46.5	9.7	NP_001078720	APE2 (ACCLIMATION OF PHOTOSYNTHESIS TO ENVIRONMENT 2); antiporter/triose-phosphate transmembrane transporter	Chloroplast	QFS#TAS#SSSFS#VK
	C65	53.5	6.36	Q6AWX0	d-xylose-proton symporter-like 2	Membrane	M*ALDPEQQQPISSVS#R
	C66	25.6	5.24	Q9SP35	Mitochondrial import inner membrane translocase subunit Tim17	Membrane	EDPWNS#IIAGAATGGFLSMR
	C67	84.6	9.29	Q9S9N5	Putative cyclic nucleotide-gated ion channel 7	Membrane	FVS#S#GETEWIK
	C68	116.2	8.17	EDQ55253.1	Na+ P-type-ATPase	Membrane	INMVY#SS#T#TVAK
	C69	98.2	5.34	NP_195035	APM1 (AMINOPEPTIDASE M1); aminopeptidase	Membrane	MLQSY#LGAEVFQK
	C70	117.7	6.91	NP_566869	CEF (clone eighty-four); protein binding/transporter/zinc ion binding	COPII vesicle coat	T#FVGIATFDS#TIHFYNLK
Cell growth/division (7.41%)	C71	137.2	6.45	EDQ50619.1	Condensin complex component SMC3	Nucleus	AEMET#QLVDS#LTPEQR
	C72	77.6	8.87	NP_850846	Kelch repeat-containing protein	Cytoplasm	S#SS#SAVLTSDES#VK
	C73	102.7	6.19	NP_567236	PMS1 (POSTMEIOTIC SEGREGATION 1); ATP binding/mismatched DNA binding	Nucleus	MQGDSS#PSPTT#TS#SPLIR
	C74	50.3	6.58	NP_199003	POLA3; DNA primase	Unknown	MDVDDDDS#DS#SLLGK
	C75	58.0	9.61	P40602	Anter-specific proline-rich protein APG	Unknown	NDIDSYT#TIIADSAAS#FVLQLY#GYGAR
	C76	82.7	7.60	NP_194589	NPGR2 (no pollen germination related 2); calmodulin binding	Membrane	DY#NGSSALST#AES#ENAK
	C77	34.5	6.76	NP_564689	Embryo-abundant protein-related	Unknown	FDT#NCVVT#TLK
	C78	16.3	9.38	NP_564332	SAUR68 (SMALL AUXIN UPREGULATED 68)	Unknown	S#NVFTS#SSS#TVEK
Cell structure (9.26%)	C79	108.8	8.62	NP_198947	Kinesin-like protein	Microtubule associated complex	INVLETLASGT#TDENEVR
	C80	98.7	9.34	Q9FJX6	Formin-like protein 6	Membrane	EEVS#EALT#DGNPESLGAELLET#LVK
	C81	80.1	8.41	NP_189792	Myosin heavy chain-related	Unknown	ET#TGSVPFPS#VGGSR
	C82	106.3	5.60	NP_567048	VLN3 (VILLIN 3); actin binding	Unknown	LY#S#IADGQVESIDGDLSK
	C83	56.8	11.32	NP_566340	Proline-rich family protein	Unknown	GTSPSPTVNS#LS#K
	C84	20.0	6.16	Q9SIX3	Putative glycine-rich RNA-binding protein	Unknown	GGMVGGYGS#GGY#R
	C85	128.9	8.51	NP_194467	VIIIB; motor	Chloroplast	Y#S#QDLIY#SK
	C86	89.2	7.24	NP_192428	ATK5 (ARABIDOPSIS THALIANA KINESIN 5); microtubule motor	Mitochondrion	T#LM*FVNISPDPS#STGESLCSLR
	C87	82.9	4.84	Q8L838	Conserved oligomeric Golgi complex subunit 4	Membrane	SCLSELGELS#S#T#FK
	C88	135.5	6.51	NP_174461	Actin binding	Unknown	S#GLS#TEMVYSMS#DK
Protein destination and storage (6.48%)	C89	75.8	8.99	NP_187752	DNAJ heat shock *N*-terminal domain-containing protein/cell division protein-related	Unknown	EY#VPSFNS#Y#ANK
	C90	105.5	5.55	Q9LV01	ETO1-like protein 2	Unknown	LASS#LGHVYS#LAGVS#R
	C91	28.3	9.39	NP_196816	Peptidyl-prolyl *cis-trans* isomerase CYP20–2	Chloroplast	MAT#LS#M*TLSNPK
	C92	74.0	7.57	NP_192255	Ankyrin repeat family protein	Unknown	TMWQHSNNGS#S#STS#TLASK
	C93	46.9	9.73	NP_566289	PAPA-1-like family protein/zinc finger (HIT type) family protein	Chloroplast	T#LQT#NSEAGTYTK
	C94	32.1	9.42	NP_199935	Ubiquinol-cytochrome C chaperone family protein	Mitochondrion	Y#GY#ATVAPAAADPPSQK
	C95	67.4	4.47	NP_191403	Meprin and TRAF homology domain-containing protein/MATH domain-containing protein	Unknown	ET#VDINGFVVVSS#K
Defence (5.56%)	C96	22.8	9.30	Q8LEQ3	Nectarin-like protein	Secreted protein	DS#TQIT#PEDFYFK
	C97	29.7	9.54	NP_178377	WHY3 (WHIRLY 3); DNA binding	Nucleus	FGDS#S#S#SQNAEVSSPR
	C98	145.4	5.64	NP_176378	Galactolipase/phospholipase	Unknown	IIVFS#GTFGPTQAVVK
	C99	17.8	5.01	NP_189276	Major latex protein-related/MLP-related	Unknown	MAT#SGT#Y#VTQVPLK
	C100	62.6	8.9	NP_001117315	Jacalin lectin family protein	Unknown	PSS#PHNAIVPHNNSGT#AQIENS#PWANK
	C101	48.9	6.08	O80998	Myrosinase-binding protein-like	Unknown	VFGS#ES#SVIVMLK
Protein synthesis (3.70%)	C102	55.6	9.10	NP_186876	eIF4-gamma/eIF5/eIF2-epsilon domain-containing protein	Unknown	IFT#HEIS#S#CYASR
	C103	28.4	5.44	NP_194797	MA3 domain-containing protein	Unknown	FT#LSSSS#DLTNGGDAPS#FAVK
	C104	50.9	5.35	NP_851097	HCF109 (HIGH CHLOROPHYLL FLUORESCENT 109); translation release factor/translation release factor, codon specific	Chloroplast	Y#TLAMAS#AVTN
	C105	11.2	6.01	NP_001031319	LCR82 Putative defensin-like protein 86	Membrane	CY#STPECNAT#CLHEGY#EEGK
Unknown (2.78%)	C106	56.4	5.60	NP_201248	Octicosapeptide/Phox/Bem1p (PB1) domain-containing protein	Chloroplast	FS#Y#NS#YPDSTDSSPR
	C107	21.4	5.44	NP_172268	LOB domain-containing protein 1	Mitochondrion	S#DASVATTPIISSSSSPPPS#LS#PR
	C108	30.1	9.32	NP_195061	Chloroplast inner membrane import protein Tic22, putative	Chloroplast	SGTPTTT#LSPS#LVAK

### Phosphorylation site identification

Protein phosphorylation in eukaryotes predominantly occurs on serine and threonine residues, whereas phosphorylation on tyrosine residues is less abundant. As expected, the serine and threonine phosphorylation sites were predominant among the phosphoproteins associated with the protoplasts regenerated for 4 d (Table 1). Tyrosine phosphorylation accounted for nearly 12.5% of all phosphorylation events, a proportion that is nearly identical to that reported previously for protonemata (13%) of *P. patens* (Heintz *et al.*, 2006). In contrast, for *A. thaliana*, phosphorylation on tyrosine residues was reported to constitute only 4.3% of protein phosphorylation events (Sugiyama *et al.*, 2008), and for animal cells (2–3%) and yeast (<1%), tyrosine phosphorylation is even less (de la Fuente van Bentem and Hirt, 2009). Evidently, the moss has a high ratio of tyrosine phosphorylation compared to other eukaryotes but the reason for this remains to be determined. Additionally, this work found tyrosine phosphorylation in multiple phosphorylated peptides, results that are similar to those for *A. thaliana* (Sugiyama *et al.*, 2008).

### Predicted localization and categorization of phosphoproteins

**Fig. 3.**
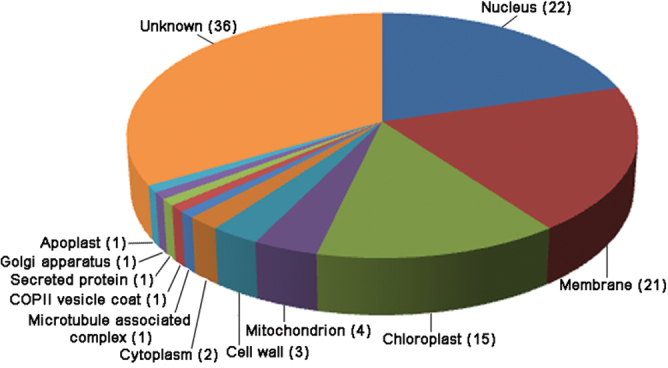
Annotated subcellular localization of phosphoproteins, showing the percentage of the 108 phosphoproteins that were specific to 4 d of regeneration in each category. Numbers in parentheses indicate the number of phosphoproteins in each subcellular localization. Localization was based on the NCBI database.

**Fig. 4.**
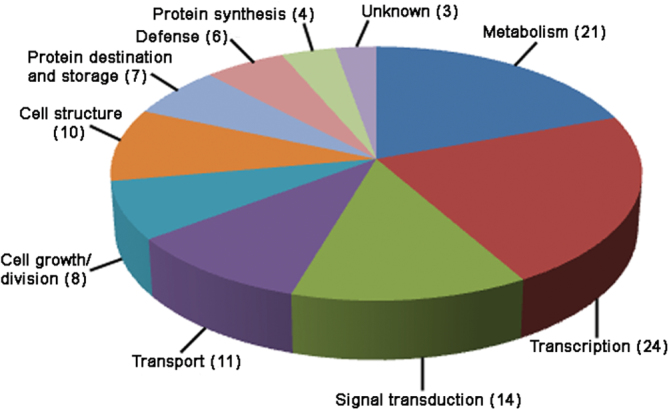
Annotated functional categorization of phosphoproteins, showing the percentage of the 108 phosphoproteins that were specific to 4 d of regeneration in each category. Numbers in parentheses indicate the number of phosphoproteins in each functional category. Function was based on the EU *Arabiposis thaliana* genome project.

The 108 phosphoproteins identified from the 4-d culture were categorized by cellular location, based on annotation within the NCBI database, and by function, based on the EU *A. thaliana* genome project (Bevan *et al.*, 1998). The majority of the proteins were predicted to be located in membrane, nucleus, and chloroplast (Table 1 and Fig. 3). As for putative function, proteins could be sorted into 10 categories (Table 1 and Fig. 4), with more than half of the proteins being involved in transcription, signal transduction, growth/division, and structure.

### Phosphoproteins involved in cell-wall metabolism and cytoskeleton structure

Among the collection of identified phosphoproteins were several involved in cell-wall metabolism, arguably one of the characteristic metabolic processes of protoplast regeneration. This work identified a xyloglucan endotransglucosylase/hydrolase (XTHs, C1) and a copper-binding oxidoreductase related to *A. thaliana* SKU5 (C2), proteins that have been previously implicated in cell-wall-loosening and expansion (Campbell and Braam, 1999; Sedbrook *et al.*, 2002). This work also identified structural cell-wall proteins, including a proline-rich family protein (C83) and a glycine-rich protein (C84) although the NCBI database does not assign either to a cell-wall location.

The identified phosphoproteins included several that are cytoskeletal. These include two members of the kinesin superfamily (C79, C86), a formin-like protein (C80), and a myosin heavy chain (C81). Kinesins are microtubule-based motors, formin is involved in the organization of the actin cytoskeleton, and myosin is a motor protein that drives actin-dependent motility.

### Phosphoproteins involved in signal transduction

At 4 d after protoplast formation, 14 proteins were categorized within the signal transduction group. Among these proteins, four are heterodimeric serine/threonine protein kinases. These kinases comprise a catalytic subunit, termed cyclin-dependent kinase (CDK, C46, C47), an activating subunit (CDK-activating kinase, CAK, C49), and a cyclin (C48). In addition, two proteins are putatively involved in signalling to the cytoskeleton. One of them is a putative homologue of TAO-1 (C50), a protein that, regulates microtubule dynamics and checkpoint signalling during mitotic progression (Draviam *et al.*, 2007), and the other is annotated as a small GTPase of the Arf6/ArfB family (C51), which is well known to regulate actin cytoskeletal organization in animals and fungi (Myers and Casanova, 2008), although apparently not studied in plants.

### Phosphoproteins associated with transcription

Among the 108 phosphoproteins, 24 of them were associated with transcription. Interestingly, the putative function of many of these transcription factors developmental. These identified transcription factors include putative homologues of the following proteins: *A. thaliana* DE-ETIOLATED1 (C22), a COP1-interacting protein (C23), a histone–lysine *N*-methyltransferase (C24), a paf1 complex subunit (C25), a bromodomain-containing protein (C27), KNOTTED OF ATHALIANA2 (KNAT2) (C28), a *A. thaliana* pumillio family member (C29), a squamosa promoter binding protein-like 14 (SPL, C31), a type I MADS-domain protein (C30), two AGAMOUS-LIKE (AGL) proteins (C32, C33), ABORTED MICROSPORES (C34), and three SNF2 family proteins (C35, C36, C37).

### Phosphoproteins involved in growth and division

There are eight phosphoproteins involved in growth and division. These proteins include a putative condensin complex component SMC3 (C72), a Kelch repeat-containing protein (C72), a putative homologue of POSTMEIOTIC SEGREGATION 1 (C73), POLA3 (C74), a putative homologue of an anther-specific proline-rich protein (C75), a putative homologue of no pollen germination related 2 (C76), a protein related to embryo-abundant proteins (C77), and SMALL AUXIN UPREGULATED68 (C78).

### Quantitative real-time PCR analysis of phosphoproteins

**Fig. 5.**
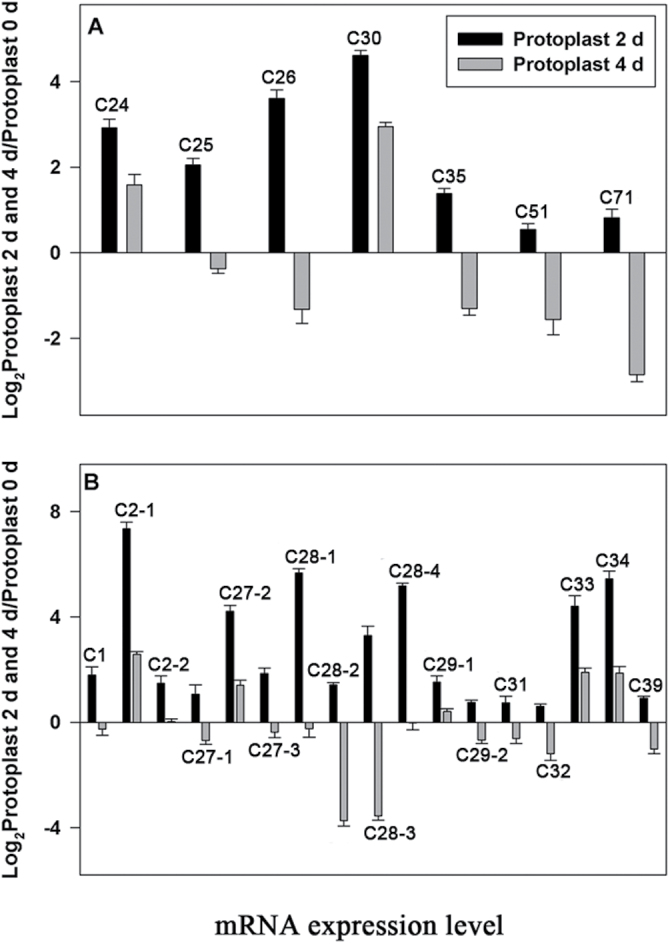
Real-time quantitative PCR, showing mRNA changed patterns of selected proteins searched for *Physcomitrella patens* protein database (A) and of the *Physcomitrella patens* homologous genes of the selected proteins searched in *Arabiposis thaliana* protein database (B). Message level is expressed as log_2_ of the ratio of the expression level at day 2 (or day 4) to day 0. All expression levels were measured relative to *P. patens* actin3 cDNA gene. Values are mean±SD of three replicate experiments.

To correlate protein level with the corresponding mRNA level, this work performed quantitative real-time PCR to analyse the mRNA expression of 24 genes of the 108 proteins specifically phosphorylated on day 4 (Fig. 5). Expression of all of the tested genes was increased at 2 d but fell at 4 d, in many cases falling substantially below the level of the fresh protoplasts. This result indicates that the transcriptional response precedes the protein phosphorylation, a result that is consistent with cellular signal transduction.

## Discussion

Two major processes that are involved in plant development are morphogenesis and organogenesis. Morphogenesis is the formation of shapes and structures, and this depends on aspects of cell behaviour such as cell-wall synthesis, cell division, and elongation. Organogenesis is the specification of organ identity. Plants are characterized by having continuous postembryonic development, where both meristematic maintenance and growth are coupled with organogenesis and reproduction (Bowman and Eshed, 2000; Gutierrez, 2005). This study shows that the mechanism of protoplast regeneration is similar to that of postembryonic development.

### Cell morphogenesis in the process of protoplast regeneration

Protoplast regeneration is accomplished by cell-wall synthesis, cytoskeleton construction, and regulation of the cell cycle. The primary cell wall consists of three coextensive polymer networks: the cellulose–xyloglucan framework, pectin, and structural protein. During protoplast regeneration, xyloglucan endotransglucosylase/hydrolase (XTH, C1), SKU5 (C2), proline-rich family protein (C83), and glycine-rich protein (C84) are examples of phosphorylated proteins that have plausible roles in primary cell-wall synthesis. In addition, squamosa promoter binding protein-like 14 (C31) is involved in cell-wall regeneration from protoplasts (Yang *et al.*, 2008).

Microtubules and actin filaments are essential components of the machinery required for nuclear division and cytokinesis. Cytokinesis in plant cells is achieved through the construction of a new cell wall between daughter nuclei after mitosis. This process is directed by the phragmoplast, a cytoskeletal structure that is made up in part by parallel microtubules and actin filaments. This work identified several phosphorylated proteins that are implicated in regulating the cytoskeleton, including TAO-1 (C49), a Arf6/ArfB family small GTPase (C50), two kinesins (C79, C86), and a Kelch repeat-containing protein (C72) that has been reported to influence cell shape through the actin cytoskeleton (Adams *et al.*, 2000), and a formin-like protein (C80) and myosin heavy chain (C81) that have been implicated in tip growth in moss (Vidali and Bezanilla, 2012). It indicates that that these cytoskeletal proteins are involved in cell division during protoplast regeneration.

Additionally, this work found several cell-cycle-regulating proteins to be phosphorylated specifically at day 4, including two cyclin-dependent kinase catalytic subunits (CDK, C46, C47), a CDK-activating kinase (C49), and a cyclin (C48). The catalytic subunits do not act alone: their ability to trigger cell-cycle events depends completely on associated cyclin subunits. The timing of activation of the CDK is be controlled by the timing of expression of a particular cyclin subunit, which also contributes to substrate specificity (Harper and Adams, 2001), and by phosphorylation. CDK-activating kinase is such an enzyme that phosphorylates CDKs to activate them (Umeda *et al.*, 2005). These versatile enzymes form the core of the cell-division cycle. Given that more than 90% of the protoplasts divide in the days after protoplast formation, it is not surprising to see evidence of protein phosphorylation among cell-cycle regulators.

### Development adjustment in response to protoplast regeneration

In this study, there were several phosphoproteins associated with development and closely related to protoplast regeneration. DET1 (C22) and COP1 control the transcription of multiple genes involved in photomorphogenesis by regulating chromatin conformation (Lau and Deng, 2012). COP1-interacting protein-related (C23) together with COP1 mediates gene expression during photomorphogenesis. Histone–lysine *N*-methyltransferase (ATX1, C24) is a chromatin modifier that trimethylates lysine 4 of histone H3 of associated nucleosomes. Histone H3 methylation affects DNA methylation and chromatin structure in ways that are consequential for development (Tamaru and Selker, 2001; Jackson *et al.*, 2002). Pumilio (C29) is a founder member of an evolutionarily conserved family of RNA-binding proteins that play an important role in embryo development, differentiation, and asymmetric division (Spassov and Jurecic, 2002).

Surprisingly, this study identified a number of transcription factors that are well known from studies of flowering in seed plants. FLOWERING LOCUS C (FLC) is a MADS-box transcriptional regulator controlling flowering time (Michaels and Amasino, 1999). *FLC* transcription is controlled in part by the PAF1 complex (C25), which mediates histone methylation of FLC chromatin (Yu and Michaels, 2010). AGAMOUS-LIKE (AGL, C32, C33) is also a MADS-box protein required for the normal development of the internal two whorls of the flower (Mizukami *et al.*, 1996). The specific function of these flowering genes in *P. patens* should be further studied.

Plant organs are formed continuously during development from meristems. ATX1 (C24) is required for the expression of homeotic genes involved in flower organogenesis (Alvarez-Venegas *et al.*, 2003). Within the meristem, the family of *KNOX* (*KNOTTED* homeobox) genes plays a crucial role in regulating organogenesis of meristematic cells (Reiser *et al.*, 2000). In *A. thaliana*, *KNAT2* (*KNOTTED*-like from *A. thaliana* 2; C28) homeobox gene is expressed in the vegetative apical meristem. It is also active during flower development and plays a role in carpel development (Pautot *et al.*, 2001). In *P. patens*, KNOX2 acts to prevent the haploid-specific body plan from developing in the diploid plant body (Sakakibara *et al.*, 2013).

Several proteins related to chromosome stability and DNA repair were also identified. The SNF2 family of proteins (C35, C36, C37) plays roles in processes such as transcriptional regulation, maintenance of chromosome stability during mitosis, and various aspects of repairing DNA damage (Eisen *et al.*, 1995). The condensing-complex component SMC3 (C71) is the core component of the tetrameric complex cohesin, which is required for the establishment of sister chromatid cohesion during S phase, maintenance of cohesion, and segregation of chromosomes in mitosis. PMS1 (C73) is a protein involved in the mismatch repair process after DNA replication (Nicolaides *et al.*, 1994). These findings suggest that these proteins play a role in protecting cell stability during protoplast regeneration.

**Fig. 6.**
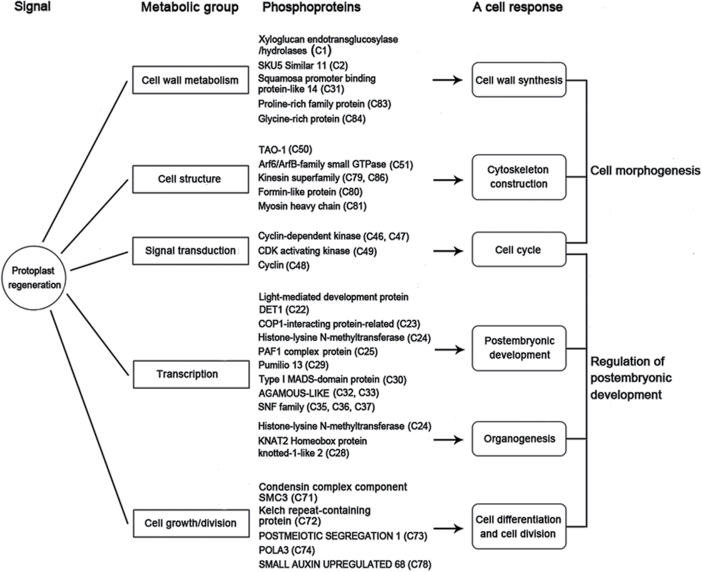
Cell responses corresponding to phosphoproteins identified during protoplast regeneration in *Physcomitrella patens*.

In conclusion, this study is, as far as is known, the first reported assessment of the phosphoproteome during of protoplast regeneration. A comprehensive analysis of the phosphoproteome involved in protoplast regeneration is presented (Fig. 6). This study indicates that there are similar mechanisms for plant protoplast regeneration and postembryonic development. Further studies of how these proteins direct the specific processes will provide deeper insight into plant protoplast regeneration.

## Supplementary material

Supplementary data are available at *JXB* online.

Supplementary Table S1. Quantitative real-time PCR primer pairs for phosphoproteins in the *P. patens* protein database.

Supplementary Table S2. Quantitative real-time PCR primer pairs for phosphoproteins in the *A. thaliana* protein database homologous to genes in the *P. patens* database.

Supplementary Data
