# Supplementary material for: The phosphoproteome in regenerating protoplasts from Physcomitrella patens protonemata shows changes paralleling postembryonic development in higher plants
Source: J Exp Bot. 2014 Apr 3;65(8):2093–106. doi: 10.1093/jxb/eru082 (PMC3991745; doi:10.1093/jxb/eru082)
Supplement: Supplementary Data [file supp_65_8_2093__index.html]

The phosphoproteome in regenerating protoplasts from Physcomitrella patens protonemata shows changes paralleling postembryonic development in higher plants — Supplementary Data 

# The phosphoproteome in regenerating protoplasts from *Physcomitrella patens* protonemata shows changes paralleling postembryonic development in higher plants

## Supplementary Data

Data files

**Files in this Data Supplement:**

- Supplementary Data - Supplementary Data
