# Supplementary material for: The phosphoproteome in regenerating protoplasts from Physcomitrella patens protonemata shows changes paralleling postembryonic development in higher plants
Source: J Exp Bot. 2014 Apr 3;65(8):2093–106. doi: 10.1093/jxb/eru082 (PMC3991745; doi:10.1093/jxb/eru082)
Supplement: Supplementary Data [file supp_eru082_eru082_Supplementary_data_v2.pdf]

The phospho-proteome in regenerating protoplasts from *Physcomitrella patens* protonemata shows changes paralleling postembryonic development in higher plants

Xiaoqin Wang,[1,2] Yong Hu,[2] Meiyan Qi,[2] Zhongzhong Ji,[2] Ramamurthy Mahalingam,[3] and Yikun He[2]*

Supplementary Table S1. Quantitative real-time PCR primer pairs for phosphoproteins in the *P. patens* protein database.

**Protein no.**	**Forward Primer**	**Reverse Primer**
C24	5"- CAGTAGTAACGGGAATATGTGGT	3"- TGAAGTAAGATGCCTTGAACAGAC
C23	5"- CTCGCCGGTATCAAGATCAGT	3"- TTCCTTAGCAGGTCCTTCAGT
C26	5"- AGTGAAGGCTCGGTTGCGTA	3"- CGAGTCTATAACCGAACAACTACA
C30	5"- TGAACTTGGGTGGAGGAATCT	3"- CGTCCACCACTACAACACCA
C35	5"- ATTCTGAAACCGCTGATGGC	3"- TCTGGCGTGTTCTAATTCCCTG
C51	5"- CCGAAGAGGCAGCTCGCTA	3"- TGTTGTCGCAGCAGAATGGC
C71	5"- ATTTTCTCGGTCAAACCCCTAC	3"- AAGTCGTCTAGTTCTGCGAGTA
Actin3	5"-GATTCAGGCAGTGTTGTCGCT	3"-TGAACTGCCTGCGTGACTAC

Supplementary Table S2. Quantitative real-time PCR primer pairs for phosphoproteins in the *A. thaliana* protein database homologous to genes in the *P. patens* database.

Protein no.	Accesession no.	Forward Priner	Reverse Primer
C1	EDQ61349.1	5"- CGATTTGTTTCTATTTGGGAGC	3"- CTTACATTCCGACTGGTCTACT
C2-1	EDQ58001.1	5"- AGTGCTTCACCGTTGGGAGT	3"- CACTACTCCTCCCCAAGCAG
C2-2	EDQ63902.1	5"- CCTGGGACGGCATACAACA	3"-GAACATCCAGGTCAAGGTTACAC
C27-1	EDQ62932.1	5"- GCTTGAACAGTATGATGGGTCG	3"- TTACTGAACTCTTGAGCCCTCT
C27-2	EDQ80839.1	5"- GGTGGGCGTTGTTTGGAT	3"- TTGGTACAGTTGGTGAGAGGAGA
C27-3	EDQ58132.1	5"- ATGGAGTATTTTCGGAGCCTGT	3"- ATGTAGCACGGCTTCAGGTA
C28-1	EDQ56932.1	5"- TGGTTCTTGCCCATGTCAGA	3"- CTTTGAAGGAGCAGTCCAGTAGAT
C28-2	EDQ76570.1	5"- GACAGAAGATGAGGACGACGC	3"- CTACTATCGTCGGGCTTCG
C28-3	EDQ62437.1	5"- TTTGGTCCTCTGGTGCCTACT	3"- TAAGAACGAGACATAGGGACGA
C28-4	EDQ53752.1	5"- GTCCGAGACTGGGCAACAT	3"- CCAGACCTATTGACCACGCT
C29-1	EDQ80534.1	5"- CCCAGCGAATGGAAATCTTAC	3"- GCATTATTGGTATCAGTGGACGAG
C29-2	EDQ75057.1	5"- CTCAGTCACGAAGACAGCCAGT	3"- TAGATGCGTAGCGACGTAGT
C31	EDQ73960.1	5"- AAAGGTCGCATTCTGGTTCA	3"- GTCACTACAAGCGACGAACTA
C32	EDQ61678.1	5"- CGGTTTGCCAACTTACCAAT	3"- GAAGGGCATTCCTCAACTACTA
C33	EDQ61137.1	5"- CAAAGGCAAATGCTTGGTGA	3"- CGACCAAGAAGGAACGAGCA
C34	EDQ62032.1	5"- ACTGGGTGTTGTTGTAGCGG	3"- GTAGACCCGTCGGACAACTC
C39	EDQ70391.1	5"- TCTATCAAGGCCGTGCTCCA	3"- GGCAGCAGTTCCGCTTTGA
Actin3	AAQ88110.1	5"-GATTCAGGCAGTGTTGTCGCT	3"-TGAACTGCCTGCGTGACTAC
